# Case Report: Novel variants in the *MYD88* gene (c.104T>C, c.141G>C) in a patient with recurrent abscesses as a cause of type 68 immunodeficiency

**DOI:** 10.3389/fimmu.2025.1683892

**Published:** 2025-12-17

**Authors:** Anastasiia A. Buianova, Artem A. Ivanov, Vera A. Belova, Alina F. Samitova, Tatiana V. Kulichenko, Iuliia A. Vasiliadis, Dmitriy O. Korostin, Yulia S. Lashkova

**Affiliations:** 1 Genomics Laboratory, Institute of Translational Medicine, Pirogov Russian National Research Medical University, Moscow, Russia; 2 Pediatric Diagnostic Department, Russian Children’s Clinical Hospital, Moscow, Russia; 3 Department for Implementation of the Functions of the National Medical Research Center for Pediatrics, Pirogov Russian National Research Medical University, Moscow, Russia

**Keywords:** fever, immunodeficiency, MyD88, pediatrics, recurrent abscesses

## Abstract

**Background:**

Primary immunodeficiencies (PIDs) comprise a heterogeneous group of disorders characterized by defects in the immune system, predisposing patients to recurrent and severe infections. Type 68 immunodeficiency, caused by biallelic pathogenic variants in *MYD88*, is rare; to date, at least 26 affected individuals have been reported in the literature, several of whom belong to the same families. This condition typically presents in early childhood with recurrent severe bacterial infections (SBIs), often accompanied by an absent or attenuated inflammatory response.

**Case presentation:**

We report a 3-month-old male patient admitted with multiple SBIs, including left-sided abscessing pyelonephritis, pyogenic liver abscess, and septic pneumonia complicated by tension pneumothorax. Initial immunological screening revealed normal leukocyte counts, immunoglobulin levels, lymphocyte subpopulations, and TREC (T-cell receptor excision circle)/KREC (kappa-deleting recombination excision circles) copy numbers. Congenital urinary tract anomalies were excluded. Despite clinical improvement, the patient subsequently developed a cold abscess of the cervical lymph node due to *Staphylococcus aureus*. Whole-exome sequencing identified two novel compound-heterozygous missense variants in *MYD88* (p.Leu35Pro and p.Trp47Cys), both located in the death domain. *In silico* analysis suggested potential disruption of α-helical structure and MyD88–MyD88/IRAK4 interactions. Sanger sequencing confirmed parental heterozygosity, establishing the diagnosis of type 68 immunodeficiency. Prophylactic antibiotic therapy was initiated, and no further SBIs occurred during 8 months of follow-up.

**Conclusion:**

This report expands the genetic spectrum of immunodeficiency 68 by identifying novel *MYD88* mutations. Our findings highlight the value of genetic testing in severe, recurrent bacterial infections, irrespective of conventional laboratory results, and demonstrate improved outcomes achievable with modern management.

## 1 Introduction

Primary immunodeficiencies (PIDs) represent a heterogeneous group of disorders characterized by defects in specific components of the immune system, resulting in an increased frequency and severity of infectious, autoimmune, and oncological diseases (1). The epidemiological profile of PIDs is characterized by substantial geographic heterogeneity. Data from the United States indicate an incidence as high as 1:1,200 (2), while global estimates average approximately 1:3,000 annually (3). Reports from several national registries have revealed a worldwide prevalence range of 1:8,500 to 1:100,000, with antibody deficiencies representing the most common category (up to 51.9% of cases) (4).

Diagnosing immunodeficiencies in childhood is particularly challenging for pediatricians due to several factors: PIDs may present at any age (from the neonatal period to adolescence); they are characterized by highly variable clinical presentations; and in infants under 3–6 months of age, there is an increased susceptibility to severe bacterial infections (SBIs) even in the absence of PID due to the immaturity of the immune system (1).

Given the complexity of PID diagnosis in childhood, several clinical tools have been proposed to assist early recognition. Among them, the “10 warning signs” formulated by the Jeffrey Modell Foundation remain widely used as a practical screening aid for identifying children who may require further immunological evaluation. Their utility has been supported by numerous studies demonstrating acceptable diagnostic performance and favorable receiver operating characteristics in diverse clinical settings (5, 6). However, a recent retrospective analysis of 2,851 children showed that 20.4% of patients with confirmed PID, including more than 20% with severe forms, exhibited none of the original 10 signs. In this cohort, an expanded set of 14 warning signs improved detection rates, with hemato-oncological disorders emerging as the strongest predictor of PID. Thus, while the 10 warning signs remain clinically useful, their interpretation should be complemented by broader clinical assessment (7).

In many developed countries, including Russia, neonatal screening using TREC/KREC quantification has been implemented to detect severe combined immunodeficiency (SCID) and other profound T- and B-cell lymphopenias in the first days of life. This approach enables pre-symptomatic diagnosis and early life-saving interventions for these specific forms of inborn errors of immunity (8).

Type 68 immunodeficiency represents a rare primary immunodeficiency disorder characterized by early-onset SBIs, typically manifesting before 2 years of age. Affected patients demonstrate particular susceptibility to respiratory and central nervous system infections caused by typical pathogens, including *Streptococcus pneumoniae*, *Staphylococcus aureus*, and *Pseudomonas* species. The clinical spectrum encompasses recurrent upper respiratory tract infections, pharyngitis, lymphadenitis, bacterial pneumonia, and bacterial meningitis. Characteristic immunological features include an absent humoral response—manifested by attenuated fever response and low C-reactive protein levels—along with invasive bacterial infections and recurrent abscess formation. Epidemiological data indicate early disease onset, high mortality before age 8 years, and no sex predilection. Laboratory evaluation may reveal neutropenia despite normal immunoglobulin and lymphocyte levels, alongside cytokine production defects. Current management strategies emphasize prophylactic antibiotics and intravenous immunoglobulin replacement therapy. Notably, fungal infections remain uncommon in this disorder (9).

In this novel case of type 68 immunodeficiency described herein, we illustrate some of the complex aspects of diagnosing PID in infants, as SBIs in this age group may result from congenital anomalies or develop independently due to immune system immaturity in the absence of PID.

## 2 Case description

The patient was born at 39 weeks of gestation via spontaneous vaginal delivery during the mother’s fourth uncomplicated pregnancy. The mother had children from two different partners (pregnancies I and II from the first partner, and pregnancies III and IV from the second). Birth parameters were within normal ranges: weight 3,400 g, length 56 cm, and Apgar scores of 8 and 9 at 1 and 5 minutes, respectively. The neonatal period was unremarkable. Physical and psychomotor development progressed normally until 3 months of age. The parents and sister had no history of recurrent or severe infections, autoimmunity, or malignancies.

**Figure 1:**
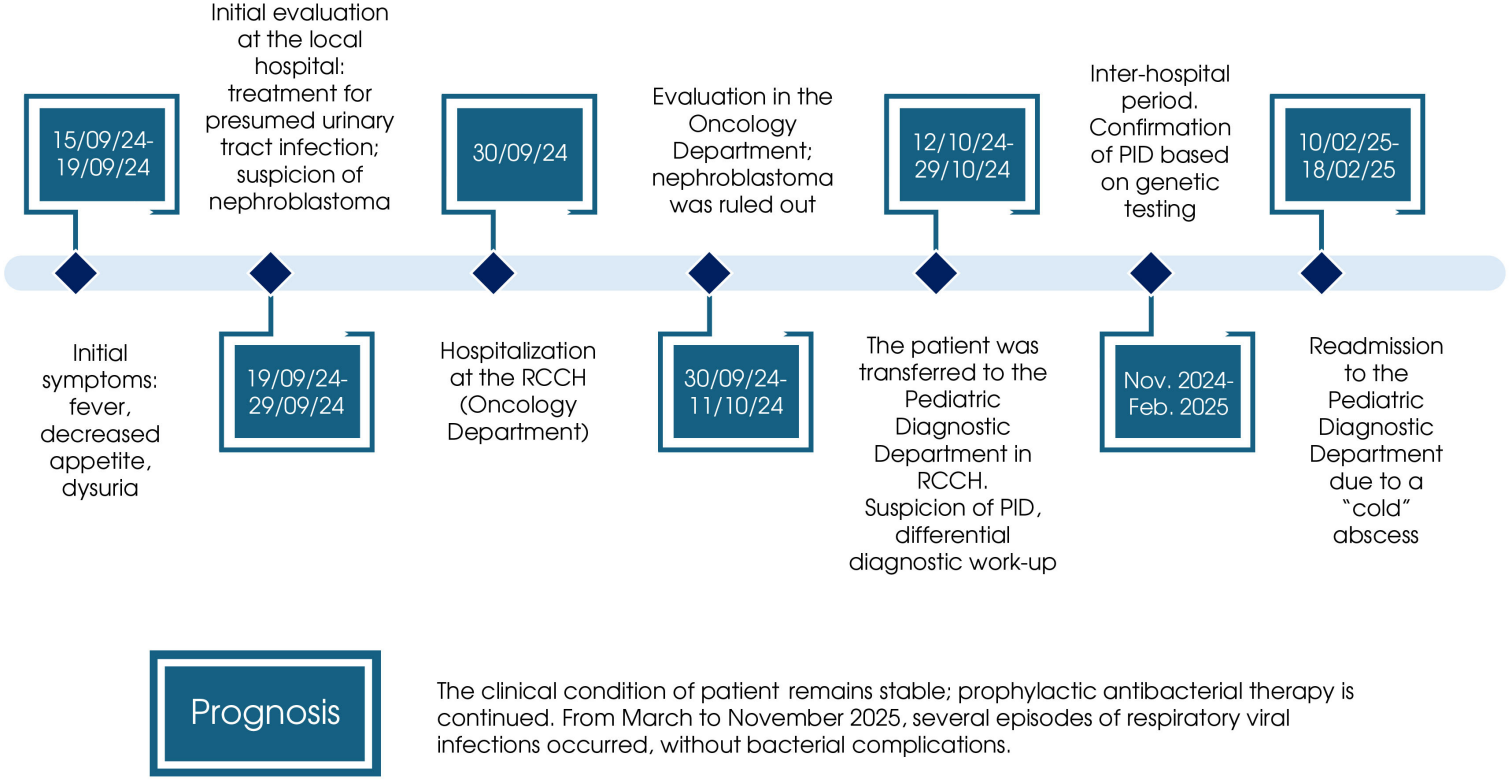
Clinical course and diagnostic workup of patient. The diagram summarizes the sequence of key events, from initial presentation with non-specific symptoms through the exclusion of nephroblastoma, the suspicion and subsequent genetic confirmation of primary immunodeficiency (PID), and the subsequent clinical course, including a readmission for a “cold” abscess. RCCH, Russian Children’s Clinical Hospital.

*Disease history* (**Figure 1**): The disease onset occurred on September 15, 2024, with the appearance of fever. The patient was evaluated by a pediatrician, who diagnosed a respiratory infection. Over the following 4 days, the patient experienced persistent fever, worsening appetite, and discomfort during urination. Due to clinical deterioration, the patient was taken to the emergency department. Blood tests revealed leukocytosis, and urinalysis indicated leukocyturia, raising suspicion of a urinary tract infection. The patient was hospitalized in the pediatric department, where a renal ultrasound suggested a renal mass (possible nephroblastoma). Antibacterial therapy led to clinical improvement and resolution of fever, and the patient was discharged for outpatient follow-up.

**Figure 2:**
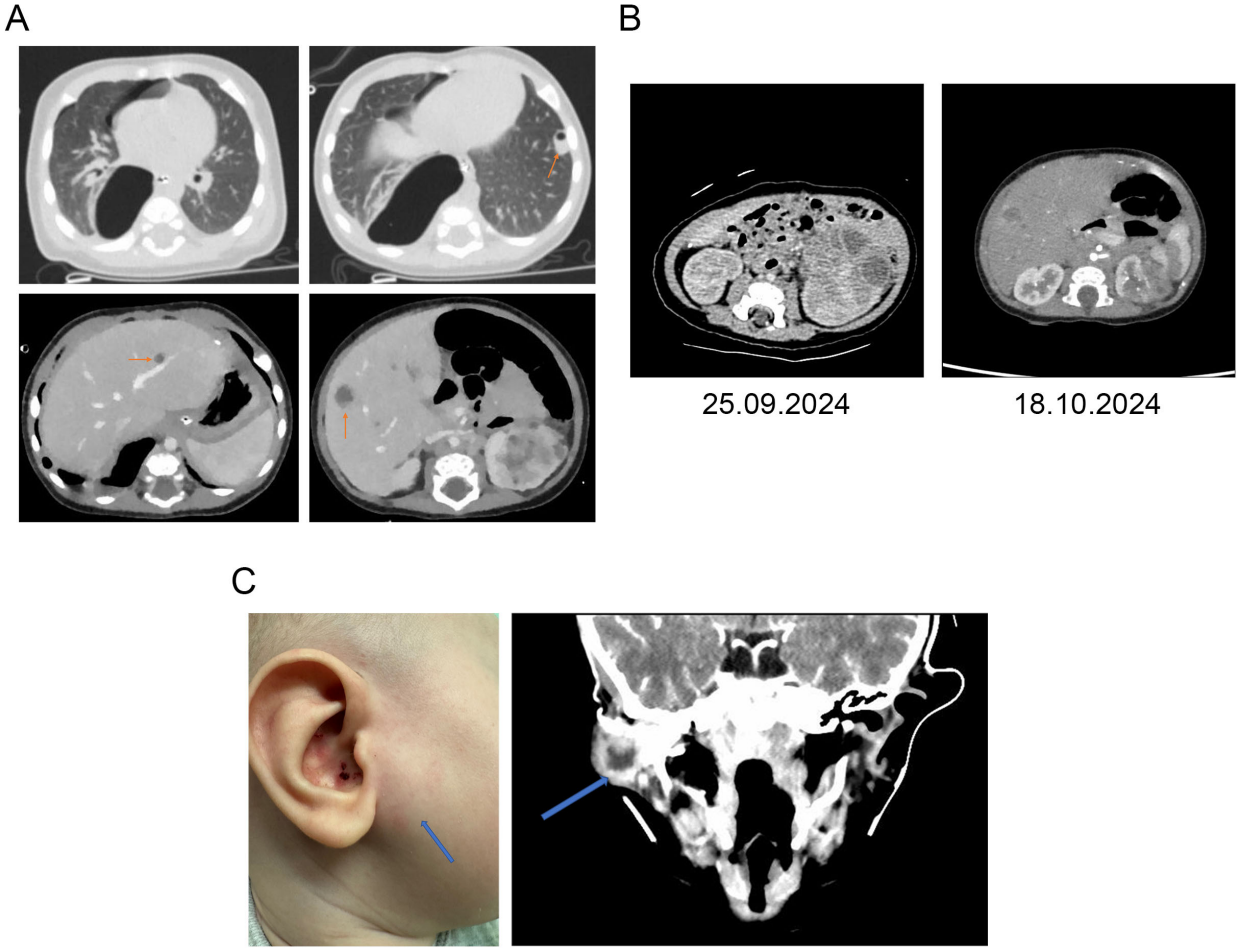
Radiological findings of patient. **(A)** CT scan of thoracic and abdominal organs. **(B)** CT of kidneys over time. **(C)** External view of cervical lymph node abscess and cervical CT scan.

However, on September 28, 2024, the patient’s condition worsened again, characterized by refusal of food and fluids. The subsequent day, he developed dyspnea accompanied by a drop in oxygen saturation to 86%, necessitating urgent hospitalization in the intensive care unit (ICU) of Russian Children’s Clinical Hospital (RCCH). In the ICU, the patient was diagnosed with a tension right-sided pneumothorax and abscess formation in the liver and lung parenchyma (**Figure 2A**).

Following pleural drainage, the patient stabilized and was referred to the oncology department for further evaluation. Antibacterial and antifungal therapy led to continued clinical improvement. Repeat renal CT showed a 50% reduction in the left renal mass (from 50 × 58 × 70 mm to 38 × 41 × 64 mm), suggestive of an inflammatory process (left-sided abscessing pyelonephritis, pyogenic liver abscess, and septic pneumonia; **Figure 2B**). No evidence of malignancy was found; the patient was transferred to the pediatric diagnostic department (by decision of the medical council) in October 2024 (**Figure 1**). At the time of admission to this department, the patient was 3 months old.

*Physical examination (upon admission to the pediatric diagnostic department)*: The patient’s general condition was satisfactory; he remained active with a good appetite. Physical examination revealed no catarrhal symptoms or visible signs of infection (no exanthema, enanthema, lymphadenopathy, diarrhea, vomiting, or urinary changes). Vital signs were within normal limits. No auscultatory abnormalities were detected in the respiratory or cardiovascular systems. No focal or meningeal signs were present.

The initial diagnoses established during the current admission to the pediatric diagnostic department were as follows: abscessing pyelonephritis; community-acquired bilateral polysegmental pneumonia, severe, complicated by right-sided pneumothorax; and pyogenic liver abscess.

Due to the occurrence of an SBI, differential diagnosis included PID and congenital anomalies of the urinary tract (vesicoureteral reflux), which could lead to pyelonephritis as a primary infection source, with subsequent systemic dissemination.

### 2.1 Clinical course and outcomes

Immunological screening revealed no abnormalities (upon admission to the pediatric diagnostic department): blood count showed no leukopenia or neutropenia (White blood cells (WBC), 9.5 × 10^9^/L; neutrophils, 3.8 × 10^9^/L; lymphocyte, 5.1 × 10^9^/L) and no hypogammaglobulinemia or lymphocyte subpopulation reduction (IgA, 0.19 g/L; IgG, 4.59 g/L; IgM, 0.81 g/L; CD3^+^, 4.2 × 10^9^/L; CD3^+^CD4^+^, 2.8 × 10^9^/L; CD3^+^CD8^+^, 1.3 × 10^9^/L; CD19^+^, 0.9 × 10^9^/L); burst test (stimulation index, 53; norm >30) and TREC/KREC levels were normal (>200 copies/100,000 leukocytes). CRP was 1.3 mg/L, and Erythrocyte sedimentation rate (ESR) was 22 mm/h, confirming a normal inflammatory response.

Cystography excluded vesicoureteral reflux, thereby ruling out congenital urinary tract pathology.

Whole-exome sequencing (WES) was recommended to exclude immunodeficiency. WES was performed on genomic DNA extracted from venous blood using QIAamp DNA Blood Mini Kit (Qiagen, Hilden, Germany), followed by MGI ultrasonic fragmentation with Covaris S220 (Covaris, Inc., Woburn, MA, USA), library preparation with MGIEasy Universal DNA Library Prep Set (MGI Tech, Shenzhen, China), and exonic sequence enrichment with Agilent All Exon v8 (Agilent Technologies, Santa Clara, CA, USA) (10). Paired-end sequencing was conducted on the G-400 platform (MGI Tech). Data analysis was performed using a Python3-based automated pipeline, including FastQC (v0.12.1), BBDuk (v38.96), BWA MEM2 (v2.2.1) for alignment, Samtools (v1.9), Picard (v2.22.4), variant calling with BCFtools (v1.9), DeepVariant (v1.5.0), vt normalize (v0.5772), InterVar (v2.2.2) for annotation, and coverage metrics (Picard v2.22.4).

WES identified two previously undescribed compound-heterozygous mutations in the *MYD88* gene (c.104T>C and c.141G>C), potentially causing type 68 immunodeficiency (OMIM: 612260). A novel heterozygous variant in exon 1 (chr3:38138804T>C, p.Leu35Pro, NM_002468.5) was not found in gnomAD (11), RUSeq (12), and was present in a heterozygous state in only one of 120,762 individuals in the FMBA database (13). *In silico* pathogenicity prediction algorithms classified the variant as pathogenic (BayesDel addAF, MetaRNN), uncertain (REVEL), or benign (MetaLR, MetaSVM).

Another novel variant (chr3:38138841G>C, p.Trp47Cys) was also identified in exon 1, not reported in any database. All prediction algorithms classified it as pathogenic.

During the inter-hospital period (15 weeks), the patient’s condition improved, with no recurrence of fever or bacterial infections. Weight and growth parameters were within normal limits.

On February 10, 2025, the patient was readmitted due to a cold abscess in the right parotid region; culture yielded *S. aureus*, further supporting a PID diagnosis (**Figure 2C**). During the hospitalization period, the patient received oral amoxicillin/clavulanic acid 600 mg + 42.9 mg at a dose of 370 mg (3.1 mL) twice daily.

**Figure 3:**
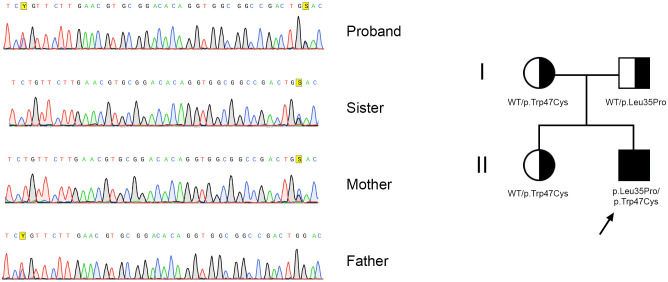
Sanger sequencing electropherograms (whole-exome sequencing validation) and family pedigree (healthy parents and sister).

Sanger sequencing of the *MYD88* gene in the patient, his sister, and parents confirmed compound heterozygosity, validating the WES results and establishing the diagnosis (**Figure 3**).

Although no specific therapy was administered, prophylactic antibacterial therapy reduced the frequency of SBI. The patient was placed on continuous oral amoxicillin prophylaxis at a dose of 20 mg/kg (165 mg) twice daily, to be maintained until the next planned hospitalization at RCCH, with dose adjustments according to weight gain under the supervision of the local pediatrician. The need for prolongation of prophylaxis was to be determined thereafter. In addition, the patient was granted a temporary medical exemption from administration of live vaccines for 6 months (until planned hospitalization at RCCH), with any future vaccinations to be performed under strict aseptic conditions.

Although most reported cases of MYD88 deficiency have been fatal in early childhood, our patient demonstrated sustained clinical improvement and no recurrent bacterial infections during an approximately 8-month follow-up period (March–November 2025), despite several episodes of viral respiratory infections.

### 2.2 *In silico* analysis of mutant proteins

**Figure 4:**
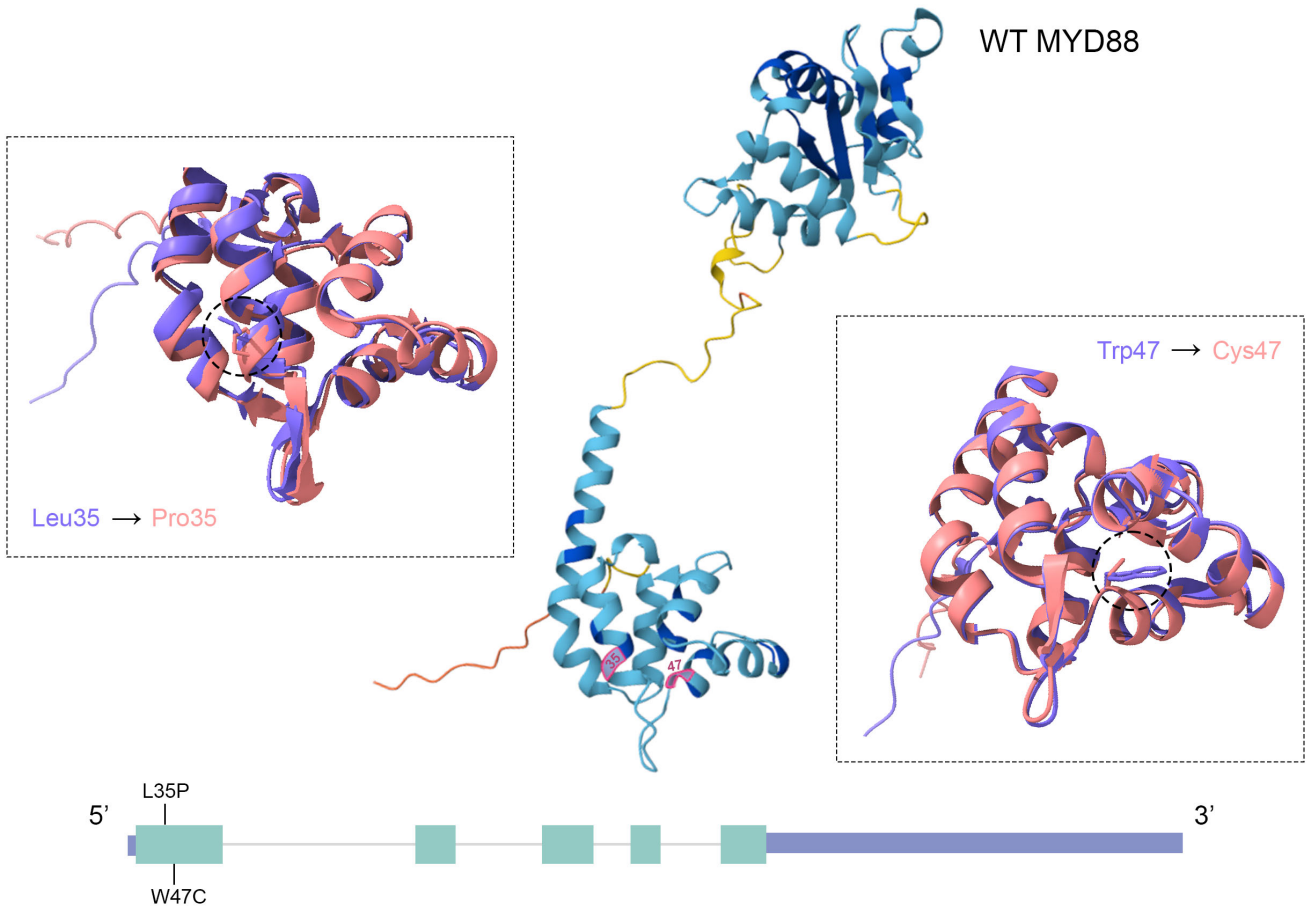
Structural alignment of WT MYD88 (purple) and mutant variants L35P and W47C (pink) using FATCAT. Structures predicted using AlphaFold3; confidence shown by Predicted local distance difference test (pLDDT) (dark blue >90, light blue 70–90, yellow 50–70, and red <50).

Mutant MYD88 proteins were modeled using AlphaFold3 (14); structural comparison was performed via FATCAT (15). Comparison between wild-type and L35P-mutant revealed 289 equivalent positions out of 292 aligned residues, with three gaps (1.03%) (**Figure 4**). RMSD was 1.49 Å, indicating high structural conservation. One twist (local bend) was detected. Sequence identity and similarity were 99.66%, FATCAT score of 837.67, and chain-Root mean square deviation (RMSD) of 12.65 Å, suggesting localized conformational changes in flexible regions.

For W47C, alignment was nearly perfect: 291 of 296 positions matched with zero gaps, with RMSD of 1.01 Å, FATCAT score of 849.23, chain-RMSD of 5.74 Å (**Figure 4**), one twist detected, and identity/similarity of 99.66%.

The Leu35Pro substitution in the death domain (DD) likely disrupts the α-helix due to proline’s rigid cyclic structure (16), potentially destabilizing MyD88–MyD88 and MyD88–IRAK4 interactions, as seen with S34Y/F mutations (17). The W47C variant also lies within the DD, potentially altering local protein structure.

## 3 Discussion

In our clinical case, after excluding oncological pathology, the primary diagnostic hypothesis was PID. However, given the specific features of the clinical presentation, we conducted comprehensive investigations to rule out alternative diagnoses. One hypothesis requiring exclusion was congenital urinary tract anomaly—specifically vesicoureteral reflux—which could have explained the development of pyelonephritis complicated by urosepsis, leading to septic emboli in the liver and lungs. Cystography excluded this possibility, thereby ruling out significant vesicoureteral reflux and raising our initial suspicion of an underlying immunodeficiency.

Additional support for this diagnosis came from the occurrence of a lymph node abscess (with isolation of a typical bacterial pathogen—*S. aureus*) that was not accompanied by fever. The diagnosis of PID was subsequently confirmed by genetic testing. It is important to note that normal results of screening tests (total immunoglobulin levels, neutrophil counts, and neutrophil subpopulation profiles) should not serve as the sole basis for ruling out PID in patients with recurrent bacterial infections. Our clinical case serves as evidence of this principle. Normal immunological screening should not preclude PID workup in patients with recurrent bacterial infections.

MYD88 is a key adaptor protein of the innate immune system involved in signal transduction from all Toll-like receptors (TLRs), except TLR3, as well as from IL-1 and IL-18 receptors. The protein consists of three functionally significant domains: the Toll/interleukin-1 receptor (TIR) domain, which mediates interaction with receptors; an intermediate domain (ID); and a DD required for the activation of IRAK family kinases. The activation of MYD88 initiates the formation of the Myddosome signaling complex, which in turn leads to the activation of the transcription factors NF-κB and AP-1 and the initiation of the inflammatory response (17).

According to crystallographic analysis of the MyD88–IRAK4–IRAK2 (DD) complex, residue Ser34 is located in the first α-helix of the DD, whereas residue Arg98 resides in the loop between the fifth and sixth helices. The substitutions analyzed in the present study—L35P and W47C—are also situated within the DD: the former in the first α-helix, near Ser34, and the latter at the beginning of the second α-helix. The S34Y and R98C mutations exhibit a dose-dependent dominant-negative effect on NF-κB activation induced by most MyD88-dependent receptors, including TLR2, TLR4, TLR5, TLR7, and IL-1R, while TLR9 remains relatively resistant to the inhibitory action of these mutations (17). Furthermore, S34Y and R98C impair the interaction between MyD88 and IRAK4, preventing the formation of a fully functional Myddosome and thereby blocking the response to pathogenic stimuli.

The two novel *MYD88* variants identified in our patient—L35P and W47C—show significant structural and potential functional parallels with previously characterized mutations. The L35P substitution, located in the first α-helix of the DD, likely exerts similar dominant-negative effects by disrupting helical structure and protein–protein interactions. The W47C variant, situated at the beginning of the second α-helix, may impair Myddosome assembly through mechanisms analogous to the R98C mutation. Both variants individually appear to be insufficient to cause disease; however, their combination in *trans* results in a pathogenic effect, consistent with the asymptomatic phenotype of the heterozygous father.

MYD88 deficiency impairs IFN-γ and IL-17 secretion by T cells (18). Known variants (e.g., E52del, L93P, and R196C) affect key domains and impair TLR signaling and IL-1β response (19). Homozygous *MYD88* E65del mutation (also known as p.Glu52del) has been linked to early SBIs (meningitis, pneumonia, and abscesses) with absent inflammatory markers (20) (**Table 1**). One patient with early-onset gastric cancer had a homozygous *MYD88* (p.Arg238Cys) TIR domain mutation, recurrent fungal infections, and partial immune defects (21). A nonsense mutation in the TIR domain of the *MYD88* gene (c.814C>T; p.Arg272Ter) results in complete loss of protein expression and is associated with severe immunodeficiency. Interestingly, in some cases, the disorder may manifest immediately after birth with delayed umbilical cord separation (up to 4 weeks) (22). In a 60-year-old patient with Waldenström macroglobulinemia, a somatic MYD88 L265P variant—present in approximately 97% of cases of this malignancy and known to activate NF-κB signaling—was identified alongside an R264* mutation previously described in the context of immunodeficiency. The detection of both variants within the tumor clone suggests that loss of function of one allele may potentiate the gain-of-function effect of the other, underscoring the dual role of MYD88 in both oncogenesis and immunodeficiency (23). A homozygous *MYD88* E52del variant has been described in a 2-year-old girl from France with SBIs (*S. aureus*, *S. pneumoniae*, *Haemophilus influenzae* type e, and *Moraxella catarrhalis*) and a fatal outcome (24). Another child from Serbia with the same homozygous variant developed invasive *Pseudomonas aeruginosa* infection but is alive. A Romanian boy with homozygous *MYD88* E52del presented with an unusually broad infectious phenotype, including peritonitis due to *P. aeruginosa*, recurrent pneumonias caused by influenza A and coronavirus NL63, mucocutaneous candidiasis, multiple viral warts, cellulitis, and *S. aureus* abscesses, as well as pneumococcal meningitis at 4 years of age. Despite severely impaired IL-8 responses and reduced effector T, Tfh, and Th17 subsets, the child survived multiple life-threatening infections and experienced COVID-19 pneumonia at age 5, underscoring the remarkably variable expressivity of MyD88 deficiency beyond classical pyogenic infections (25). A recent multicenter study further expanded the phenotype of MYD88 deficiency by reporting patients, all carrying the homozygous E52del variant, who developed hypoxemic or critical COVID-19 pneumonia due to impaired TLR7-dependent type I IFN responses. These findings demonstrate that MYD88 deficiency confers a markedly increased risk of severe SARS-CoV-2 infection, comparable to that observed in TLR7-deficient individuals (26).

**Table 1:** Comparative clinical and immunological characteristics of patients with *MYD88* gene variants.

Reference	Sex	Age	Infections	Inflammatory response	Outcome	*MYD88* gene variants
de Beaucoudrey, L. et al. (18)	F	16 years	*Pneumococcus*, *Salmonella* (Salmonella enteritidis (Se), Salmonella typhimurium (St), Salmonella dublin (Sd))	N/A	Alive	R196C
	M	10 years	*Pneumococcus*, *Salmonella* (Se, St, Sd), *Staphylococcus*	N/A	Alive	R196C
	F	4 years	*Pneumococcus*	N/A	Alive	L93P, R196C
von Bernuth, H. et al. (19)	M	10 months	Pharyngitis at 5 months (*Pseudomonas aeruginosa*), abscess in sphenoid and ethmoid sinuses, cavernous sinus, sella turcica, and both orbits at 8 months (*P. aeruginosa*, *Staphylococcus aureus*), meningitis at 10 months (*Streptococcus pneumoniae*)	Temperature <38°C, CRP < 30 mg/L, leukopenia, weak inflammatory response	Lethal outcome 4 days after meningitis onset	E52del
	F	3 years	Recurrent skin infections from 1 month, left inguinal abscess at 9 months, meningitis at 1 year 8 months (*S. pneumoniae*)	High CRP and ESR, temperature 38.5°C	Improvement, prophylaxis	L93P, R196C
	F	16 years	7 episodes of meningitis: 6 weeks, 8 months, 14 months, 16 months, 16 months, 3 years, and 6 years (*S. pneumoniae*, non-typhoidal *Salmonella*), cutaneous and subcutaneous infections	Normal inflammatory response	>10 years infection-free on prophylaxis	R196C
	M	9 years	Cervical lymphadenitis, inguinal abscess, bacteremia at 2 months (*S. aureus*, *P. aeruginosa*, *Proteus* spp.), meningitis at 4 months (group B β-hemolytic *Streptococcus*) and at 15 months (*S. pneumoniae*), lobar pneumonia at 2 years, gastroenteritis and sacroiliitis at 3 years (*Salmonella* Enteritidis), arthritis of left hip joint (*S. aureus*)	Low CRP, often afebrile	>3 years infection-free on prophylaxis	R196C
	F	11 months	Submandibular abscesses at 5 and 8 months (*S. aureus*), meningitis (*S. pneumoniae*)	Afebrile, CRP < 3 mg/L	Lethal outcome due to meningitis	E52del?
	M	3.5 years	Retroauricular lymphadenitis at 2 and 3 years (*Streptococcus pneumoniae*), submandibular lymphadenitis at 3 years 3 months, cervical lymphadenitis at 3.5 years (*S. aureus*), cellulitis, paronychia of the left big toe at 3.5 years (*S. aureus* and *Citrobacter* sp.)	Weak inflammatory response, no fever	Improvement	E52del
	M	1 month	Undocumented sepsis	N/A	Lethal outcome	E52del?
	M	7 years	Urinary tract infection (UTI) at 4 months (*Klebsiella pneumoniae*), osteomyelitis of the talus at 9 months (group C β-hemolytic *Streptococcus*), gastroenteritis at 16 months (*Campylobacter jejuni*), submandibular lymphadenitis 10 days later	No fever, CRP < 3 mg/L	Improvement	E52del
	F	4 years	Arthritis of right knee at 2 years 3 months (*S. pneumoniae*), arthritis of right ankle at 2.5 years (*S. pneumoniae*), mediastinal and mesenteric lymphadenitis at 4 years 2 months (*S. aureus*)	Maximum temperature up to 38°C, moderate inflammatory response	Improvement	E52del
Vogelaar, I. P. et al. (21)	F	23 years	Onycholysis at 2 years, recurrent vaginal infections (*Candida albicans*), dermatophytic onychomycosis	Impaired Th17 response, normal TLR function	Alive, gastric cancer	R238C
Platt, C. D. et al. (22)	M	N/A	Bacillus Calmette–Guerin (BCG) adenitis at 3 months, pneumonia at 5 months (*P. aeruginosa*), Methicillin-resistant Staphylococcus aureus (MRSA) abscess of the supraclavicular lymph node at 5 months	Impaired TNF-α and IL-6 production in response to stimulation via MyD88-dependent receptors (TLR2, TLR4, IL-1R), ↓IgM, ↓B cells, severe neutropenia	Improvement, prophylaxis	R272Ter
Gautam, A. et al. (23)	F	60 years	Waldenström’s disease	Leukopenia and neutropenia	Alive	L265P (somatic), R264*
Picard, C. et al. (24)	F	2 years	*S. aureus*, *S. pneumoniae*; *Haemophilus influenzae* type e; *Moraxella catarrhalis*	Unremarkable immunological findings	Lethal outcome	E52del/E52del
	M	1 year	*P. aeruginosa* sepsis	N/A	Alive	E52del/E52del
Bucciol, G. et al. (25)	M	3 months	Omphalitis at birth, a skin abscess on chest and peritonitis (*P. aeruginosa*) at 4 months, basilar pneumonitis at 18 months, bilateral pneumonia (coronavirus NL63 and influenza A virus), cellulitis, a cutaneous abscess caused (*S. aureus*), as well as recurrent throat infections and multiple warts (verruca vulgaris) on the face at 3 years, meningitis (*S. pneumoniae*) at 4 years, COVID-19 pneumonia at 5 years	Adequate systemic inflammatory response, IL-8 secretion was severely impaired, ↓ effector T cell, ↓T follicular helper, ↓Th17	Alive	E52del/E52del
Our patient	M	7 months	Left-sided abscessing pyelonephritis at 2.5 months, pyogenic liver abscess at 2.5 months, septic pneumonia at 2.5 months, cervical lymph node abscess at 7 months (*S. aureus*)	Fever, normal inflammatory response	Improvement	L35P, W47C

With the addition of these reports, a total of 26 patients with genetically confirmed MYD88 deficiency have now been described in the literature (**Table 1**), highlighting the rarity of this condition. Given the limited cohort size, it is challenging to define a “typical” clinical presentation. Nevertheless, several features of the current case are noteworthy:

- The patient exhibited no signs of impaired physical development, consistent with the established MYD88 deficiency phenotype (24).
- With extended follow-up spanning 8 months, the patient remains alive and has experienced only one additional serious bacterial infection (cervical lymph node abscess) while receiving prophylactic antibiotics. This sustained survival markedly contrasts with the early childhood mortality reported in prior cases (19). However, as the patient has not yet reached 2 years of age, longer follow-up is required to draw definitive conclusions about the long-term outcome.
- The patient experienced no episodes of neuroinfection, which are more commonly reported in this PID subtype (19).

## 4 Conclusion

This study characterized two novel *MYD88* DD variants (L35P and W47C) that expand the genetic spectrum of this rare immunodeficiency. Structural analyses indicated that both mutations disrupt critical protein–protein interactions essential for Myddosome assembly. The patient’s sustained survival beyond 8 months demonstrates that early molecular diagnosis and modern management can significantly improve the historically poor prognosis of MYD88 deficiency. This case emphasizes that despite inherent SBI susceptibility in young infants, recurrent invasive infections with a blunted inflammatory response should prompt genetic evaluation.

## Data Availability

Original datasets are available in a publicly accessible repository: The original contributions presented in the study are publicly available. This data can be found here: https://www.ncbi.nlm.nih.gov/sra under accession number PRJNA1307629.
